# Eicosapentaenoic Acid Improves Porcine Oocyte Cytoplasmic Maturation and Developmental Competence via Antioxidant and Mitochondrial Regulatory Mechanisms

**DOI:** 10.3390/antiox15010137

**Published:** 2026-01-21

**Authors:** Yibo Sun, Xinyu Li, Chunyu Jiang, Guian Huang, Junjie Wang, Yu Tian, Lin Jiang, Xueping Shi, Jianguo Zhao, Jiaojiao Huang

**Affiliations:** 1 College of Animal Science and Technology, Qingdao Agricultural University, Qingdao 266109, China; 19050210751@163.com (Y.S.); qdguian@sina.com (G.H.); 202101017@qau.edu.cn (J.W.); leon.tianyu@hotmail.com (Y.T.); xuepingsh@163.com (X.S.); 2 College of Life Sciences, Qingdao Agricultural University, Qingdao 266109, China; lixinyu@stu.qau.edu.cn (X.L.); jiangchunyu@stu.qau.edu.cn (C.J.); 3 State Key Laboratory of Organ Regeneration and Reconstruction, Institute of Zoology, Chinese Academy of Sciences, Beijing 100101, China; 4 State Key Laboratory of Animal Biotech Breeding, College of Animal Science and Technology, China Agricultural University, Beijing 100193, China; b20233040348@cau.edu.cn

**Keywords:** eicosapentaenoic acid, porcine oocyte maturation, antioxidant defense, mitochondrial dynamics

## Abstract

Oocytes cultured in vitro are exposed to high oxygen tension and lack follicular antioxidants, leading to redox imbalance. Eicosapentaenoic acid (EPA), a marine long-chain n-3 polyunsaturated fatty acid, possesses strong antioxidant activity. Here, using pigs as a model, we examined the effects of EPA on oocyte in vitro maturation (IVM) and subsequent developmental competence. Cumulus–oocyte complexes were cultured with EPA, followed by assessment of nuclear and cytoplasmic maturation and embryonic development; transcriptomic and proteomic analyses were conducted to explore underlying mechanisms. Supplementation with 10 µM EPA significantly improved maturation and blastocyst rates by reducing spindle defects, facilitating a more uniform organization of cortical granules and mitochondria. EPA increased resolvin E1 accumulation and reduced cumulus-cell apoptosis through downregulation of *TNF-α* and *BAX* and upregulation of *BCL2*. In MII oocytes, EPA lowered apoptosis, DNA damage, and ROS levels while enhancing *SOD2* and *GPX4* expression. Mitochondrial quality and turnover were improved via upregulation of *PPARGC1A*, *NDUFS2*, *PINK1*, *LC3*, *FIS1*, *MUL1*, and *OPA1*, alongside strengthened ER–mitochondria contacts. These findings demonstrate that EPA alleviates oxidative stress, optimizes mitochondrial function, and enhances porcine oocyte maturation and developmental competence in a parthenogenetic model, highlighting its potential as a marine-derived functional additive for reproductive biotechnology. Future studies will be required to validate these effects under fertilization-based embryo production systems and to further refine dose–response relationships using expanded embryo-quality endpoints.

## 1. Introduction

Oocytes are essential components of maternal genetic inheritance. Mammalian oocyte in vitro maturation (IVM) is critical for a variety of disciplines, including cellular research, conservation biology, animal breeding, and reproductive medicine. However, not all oocytes develop into a viable embryo post-IVM, and culture techniques can diminish gamete quality, presenting challenges for the practical application of somatic cell nuclear transfer and IVF.

The process of in vitro maturation regulates oocyte meiotic completion and later embryonic development through changes in the intracellular microenvironment [1]. For example, reactive oxygen species (ROS) increase due to oxygen tension during oocyte IVM [2]. Under typical circumstances, cells generate ROS at physiological levels beneficial for tissue regeneration, intracellular redox regulation, and embryogenesis; however, excessive ROS can oxidize cellular molecules, including lipids, carbohydrates, amino acids, and nucleic acids, altering their functions and jeopardizing cell viability through lipid peroxidation, mitochondrial impairment, apoptosis and meiotic defects [2]. The detrimental effects of excessive ROS are mitigated through the use of a diverse array of antioxidants during oocyte IVM, including Vitamin C [3], glycine [4], and nobiletin [5].

Among these bioactive compounds, long-chain n-3 polyunsaturated fatty acids (PUFAs), including alpha-linolenic acid (ALA), eicosapentaenoic acid (EPA), and docosahexaenoic acid (DHA), are predominantly derived from marine organisms such as cold-water fish and microalgae, providing a vital nutritional link between marine ecosystems and mammalian health. These fatty acids have gained increasing attention due to their abilities to alter plasma membrane components [6], exert antioxidant effects [7] and regulate cell signaling [8]. Since n-3 PUFAs cannot be synthesized endogenously in most mammals, they must be obtained from the diet. Dietary n-3 PUFAs have been shown to improve folliculogenesis and reduce pregnancy loss in both humans and animals [9,10].

Mechanistically, n-3 PUFAs function as endogenous activators of peroxisome proliferator-activated receptors (PPARs), which play pivotal roles in regulating ovarian folliculogenesis, oocyte maturation, and early embryonic development [11,12]. N-3 PUFAs also provide the precursors for prostaglandin synthesis and can modulate the expression patterns of many key enzymes involved in both prostaglandin and steroid metabolism [13]. Moreover, n-3 PUFAs act as precursors for anti-inflammatory lipid mediators. For instance, EPA can be metabolized into resolvin E1 (RvE1), a bioactive lipid mediator with potent anti-inflammatory and pro-resolving properties [14].

In vitro studies have shown that direct supplementation of n-3 PUFAs in maturation media can improve oocyte quality and developmental competence. For example, the addition of 50 μM n-3 PUFAs during IVM of oocytes from polycystic ovary syndrome mice significantly enhanced maturation rates by alleviating oxidative stress and reducing spindle and chromosome abnormalities, thereby supporting meiotic integrity [15]. Similarly, supplementation of IVM media with a combination of LA and ALA in bovine oocytes decreased ROS levels, increased total cell numbers in blastocysts, and reduced apoptosis, collectively indicating improved embryonic quality [16]. Moreover, the effects of DHA during IVM are dose-dependent. A low physiological concentration of DHA (1 μM) has been shown to enhance oocyte developmental competence in cattle without affecting the expression of lipid metabolism-related genes in cumulus cells; in contrast, exposure to a higher dose (100 μM) impaired oocyte quality, likely due to disruptions in lipid and steroid metabolism within the cumulus cell environment [17,18]. These observations underscore the necessity of optimizing both the composition and concentration of n-3 PUFA supplementation in IVM systems to support redox homeostasis while preserving metabolic balance.

Therefore, this study investigated the antioxidant regulatory effects of EPA during porcine oocyte in vitro maturation. Specifically, we sought to assess its influence on nuclear maturation, cytoplasmic maturation, and subsequent embryonic developmental competence in a parthenogenetic model. Furthermore, we explored potential mechanisms by integrating transcriptomic analysis of cumulus cells with transcriptomic and proteomic profiling of matured oocytes. Our findings demonstrate that supplementation with 10 μM EPA alleviates oxidative stress, promotes mitochondrial quality control through autophagy and biogenesis, and ultimately enhances porcine oocyte maturation and developmental potential. Collectively, our findings reveal the reproductive benefits of a marine-derived bioactive lipid and underscore the potential of marine n-3 PUFAs as functional ingredients for improving in vitro embryo production systems and advancing the utilization of marine biological resources.

## 2. Materials and Methods

### 2.1. Ethics Statement

This study was conducted in accordance with approval from the Ethics Committee of Qingdao Agricultural University (DEC2021-18).

### 2.2. Media and Reagents

Unless otherwise noted, all compounds were purchased from Sigma (St. Louis, MO, USA). A 0.22 mm filter was used to filter all of the following solutions and materials.

### 2.3. Porcine Oocytes In Vitro Maturation

The procedures for collection and IVM of porcine oocytes were carried out as described in our previous study [19]. Ovaries of gilts were obtained from a local slaughterhouse and transported to the laboratory in 0.9% saline (*w*/*v*) supplemented with 0.03 g/mL penicillin-G and 0.03 g/mL streptomycin sulfate at 30–35 °C within 3 h. Follicular fluid with the oocytes was aspirated from the antral follicles (3–8 mm in diameter) and collected into a 50 mL centrifuge tube using an 18-gauge needle connected to a 10 mL disposable syringe. Cumulus–oocyte complexes (COCs) were recovered under a stereomicroscope (Nikon Corporation, Tokyo, Japan; SMZ7800). The COCs with at least three layers of compact cumulus cells and a homogeneous cytoplasm were selected for IVM. The selected COCs were washed thrice with IVM medium. Approximately 70–80 oocytes were transferred to each well of a four-well dish (Thermo Fisher Scientific, Inc., Waltham, MA, USA) into 500 μL IVM media supplemented with different concentrations of EPA, and cultured at 38.5 °C with 5% CO_2_ for 42 h in a humidified atmosphere. The COCs were treated with 0.1% hyaluronidase and 0.01% polyvinyl alcohol (PVA) for 4 min, to remove the surrounding cumulus cells. The oocytes were observed under a stereomicroscope, and those with the first polar body in the perivitelline space were considered as mature. The mature oocytes with intact cell membranes and clear perivitelline space were selected for follow-up tests.

### 2.4. Parthenogenetic Activation and Embryo Culturing

The MII oocytes were activated electrically in a medium containing 0.3 M mannitol, 1.0 mM CaCl_2_, 0.1 mM MgCl_2_, and 0.5 mM HEPES. Between the electrodes (1.0 mm diameter), two direct current pulses (1.25 kV/cm) were applied for 30 µs at 1 s intervals using an Electro Cell Fusion Generator (Nepa Gene, Ichikawa, Japan). The electro-activated oocytes cultured in a four-well dish containing porcine zygote medium-3 (PZM-3) at 38.5 °C under 5% CO_2_. On days 2 and 6, the number of cleavage and blastocyst embryos was recorded to calculate their formation rates.

### 2.5. Morphology of Oocyte Spindle

After samples were rinsed in PBS, the MII oocytes were fixed in 4% paraformaldehyde in PBS for 15 min and permeabilized in 1% TritonX-100 in PBS for 30 min. Then the oocytes were blocked in 5% BSA in PBS for 1 h at room temperature. Then, the sample was incubated overnight at 4 °C with anti-*α*-tubulin (St. Louis, MO, USA, T6199) at a dilution of 1:1000. After extensive washing with PBS, the samples were incubated with FITC rabbit anti-goat IgG (ABclonal Biotechnology Co., Ltd., Wuhan, China, AS024) at a dilution of 1:1000 for 1 h at room temperature. Then the oocytes were treated with 10 mg/mL Hoechst 33342 (Beyotime Biotechnology, Shanghai, China, C1022) for 10 min to detect DNA. They were mounted on glass slides with antifade mounting medium (Beyotime Biotechnology, Shanghai, China; P0126) and examined using laser scanning confocal microscopy of the model Leica TCS SP5 II (Leica Microsystems, Wetzlar, Germany). For comparisons, the exposure and image capture settings were kept constant, and all images were compiled without any contrast or brightness adjustments.

### 2.6. Localization and Distribution of Mitochondria in the MII Oocyte

MII oocytes were stained for mitochondria by 0.5 mM MitoTracker Red CMXRos for 30 min at 37 °C (Meilun Biotechnology Co., Ltd., Dalian, China, MB6046). The samples were then fixed in PBS containing 2% (*w*/*v*) paraformaldehyde for 10 min at room temperature. To detect DNA, the oocytes were treated with 10 mg/mL Hoechst 33342 for 10 min. Then, they were mounted on glass slides with antifade mounting medium and examined using laser scanning confocal microscopy (Zeiss, Oberkochen, Germany). The fluorescence intensity was analyzed by ImageJ software v. 1.54r (National Institutes of Health, Bethesda, MD, USA).

### 2.7. Evaluation of Cortical Granule Distribution in the MII Oocyte

MII oocytes were first fixed with 4% (*v*/*v*) paraformaldehyde. After permeabilizing with 1% (*v*/*v*) tritonX-100 (30 min), the oocytes were stained for 30 min with 100 mg/mL FITC-PNA (St. Louis, MO, USA, L7381). Subsequently, the stained oocytes were mounted on glass slides with antifade mounting medium and examined using a confocal laser scanning microscope.

The cortical granule distribution was categorized as follows: peripheral (cortical granules were adjacent to the plasma membrane, indicating cytoplasmic maturation), cortical (cortical granules were localized in the cortical area of oocytes, indicating partial maturation), homogeneous (cortical granules were scattered throughout the cytoplasm, indicating a lack of cytoplasmic maturation), and abnormal distribution (cortical granules had an irregular distribution, indicating a poor quality or deterioration) [20].

### 2.8. Enzyme-Linked Immune Sorbent Assay (ELISA) of RvE1

Fresh porcine COCs IVM medium (0 h) and those cultured for 24 h, 30 h, 36 h, and 42 h were collected and stored at −80 °C. RvE1 concentrations were determined using Porcine RvE1 ELISA Kit (Jingmei Biotechnology Co., Ltd., Taixing, China, JM-10423P1) following the manufacturer’s protocol. Standard samples were prepared according to kit instructions. Fivefold diluted samples and standard solutions were added to ELISA plate wells, sealed, and incubated at 37 °C for 30 min. After discarding the liquid, wells were washed 5 times with 30-fold diluted wash buffer. Subsequently, 50 μL of enzyme-linked reagent was added to each well (except blanks), followed by incubation and washing as before. Then, 50 μL of reagents A and B were added, mixed, and incubated in the dark at 37 °C for 10 min. The reaction was stopped with 50 μL of stop solution, and OD values at 450 nm were measured using a microplate reader (BioTek Instruments, Winooski, VT, USA). Sample concentrations were calculated based on a standard curve derived from linear regression.

### 2.9. ROS Level Detection

The ROS levels in the oocytes were measured using the ROS assay Kit (Beyotime Biotechnology, Shanghai, China, S0033S) according to the manufacturer’s instructions. Briefly, the oocytes were incubated with 100 M 2,7-dichlorodihydrofluorescein diacetate in M199 medium at 38.5 °C for 15 min in the dark. After washing three times with M199 medium containing 3 mg/mL bovine serum albumin according to the manufacturer’s instructions at room temperature, the oocytes were transferred into 30 µL PBS droplets, and the fluorescence was observed using a fluorescence microscope (Nikon, Tokyo, Japan).

### 2.10. Annexin-V Analysis

According to the manufacturer’s instructions, to detect early apoptosis in oocytes using the Annexin V-FITC Apoptosis Detection Kit (Beyotime Biotechnology, Shanghai, China, C1062S), the washed oocytes were placed in 195 μL of Binding Buffer, followed by the addition of 5 μL of Annexin V-FITC. The mixture was gently mixed and incubated in the dark at room temperature for 15 min. The oocytes were then transferred to a 30 µL droplet of PBS for observation under a fluorescence microscope (Eclipse Ti2, Nikon, Japan).

### 2.11. γ-H2AX Detection

Mature oocytes were rinsed with PBS, fixed in 4% paraformaldehyde in PBS for 15 min, and permeabilized in 1% TritonX-100 in PBS for 30 min. Then they were blocked with 5% BSA for 1 h at room temperature. According to the manufacturer’s instructions, the oocytes were incubated with primary antibodies against γ-H2AX (1:1000, Abcam, Cambridge, MA, USA ab88770) overnight at 4 °C. After being washed three times with PBS, the samples were treated with the secondary antibody, Goat Anti-Rabbit lgG AF 488 (1:1000, Abmart Shanghai Co., Ltd., Shanghai, China, M21012) in the dark for 1 h at room temperature. Then the oocytes were treated with propidium iodide (PI; Beyotime Biotechnology, Shanghai, China, C1352) for 8 min to detect DNA. They were mounted on slides and examined by laser scanning confocal microscopy. For comparison, the exposure and image-capture settings were kept constant, and all images were compiled without any contrast or brightness adjustment.

### 2.12. Assessment of Cumulus Cell Expansion

Cumulus cell expansion was graded based on previous experience [21]. Briefly, the expansion of cumulus cells were divided into three classes, first, poorly expansion (the cumulus cells is tightly wrapped around the oocyte); second, partially expansion (the outermost cumulus cells of COCs expand radially, and the diameter of COCs can be up to twice that of oocyte); third, full expansion (the diameter of the COCs exceeded three times that of the oocyte).

### 2.13. RNA-Seq and Analysis

Cumulus cells and MII oocytes derived from the control and 10 μM EPA-treatment group were collected and immediately frozen at −80 °C. RNA samples with high purity (OD 260/280 ≥ 2.0) and high integrity (RIN > 7) were used for cDNA library construction. Sequencing library preparation and RNA-seq were conducted at LC-Bio Technology Co., Ltd. (LC-Bio, Hangzhou, China). Fold change (FC) ≥ 1.5 or FC ≤ 0.67 (i.e., absolute value of log_2_FC ≥ 1.5) and padj < 0.05 were used as thresholds for the detection of differentially expressed genes (DEGs). For DEGs list, GO enrichment analysis has been separately carried out using the “clusterProfiler”. Ligand–receptor pair activity between cumulus cells and oocytes utilized the Ensembl Biomart tool to map differentially expressed genes to their human orthologs. Ligand–receptor pair information was subsequently retrieved from the CellChatDB database, and ligand–receptor pairs were filtered based on the set of differentially expressed genes. To quantify the potential activity of each ligand–receptor pair, an activity score was calculated using the formula:

$$L-R\;Score=\sqrt{{TPM}_{Ligand}\times {TPM}_{Receptor}}$$

### 2.14. Tandem Mass Tags (TMT) Quantitative Proteomics Analysis

We obtained 1500 MII stage oocytes from both the control and 10 μM EPA-treated groups, with 500 oocytes per sample, including three samples for each group. TMT quantitative proteomics technology was performed at Novogene Biotechnology Co., Ltd. (Novogene, Beijing, China), including protein extraction, protein quality check, proteolytic desalting, peptide labeling, fraction separation, liquid phase detection, mass spectrometry detection, and data analysis. Fold change (FC) ≥ 1.5 or FC ≤ 0.67 (i.e., absolute value of log_2_FC ≥ 1.5) and *p* < 0.05 were used as thresholds for the detection of differentially expressed proteins (DEPs). For DEPs list, pathway and process enrichment analysis has been separately carried out using the “Metascape” Gene Annotation and Analysis Resource.

### 2.15. Real-Time Quantitative PCR Analysis

Total RNA was extracted from the oocytes or GCs using AG RNAex Pro Reagent (Accurate Biology, Changsha, China, AG21102) according to the manufacturer’s instructions. cDNA was synthesized using the SPARKscript II RT Plus Kit (Sparkjade, Jinan, China, AG0304). The CFX96 Real-Time System (Bio-Rad, Hercules, CA, USA) was used to perform real-time PCR using the SYBR Green mix. The expression of the pig *GAPDH* acted as an internal control (primer sequences are shown in Table S1). The 2^−ΔΔCt^ method was used to calculate the fold changes in gene expression and all experiment runs were repeated in three replications.

### 2.16. Transmission Electron Microscopy Analysis

Mature oocytes were examined utilizing transmission electron microscopy (Hitachi, Ltd., Tokyo, Japan). Initially, a total of 30 oocytes from both the control and experimental groups, with three biological replicates for each, were collected and fixed in 2.5% glutaraldehyde at 4 °C overnight. The samples were subsequently fixed in 1% osmium tetroxide for a duration of 2 h, underwent a gradient dehydration process, and then infiltrated with resin and embedded in molds for polymerization. Ultra-thin sections of the samples were prepared and subjected to double-staining with 2% uranyl acetate and lead citrate. The samples were ultimately observed under an electron microscope. For each oocyte, at least two thin sections were cut, and a minimum of five distinct oocyte sections from each group were analyzed. Three random areas of the oocytes were selected for observation at a magnification of 1.2 k×, and the ratio of mitochondrial aggregates surrounded by the endoplasmic reticulum to the total number of mitochondria was statistically evaluated.

### 2.17. Statistical Analysis

All experiments were performed with at least three independent biological replicates, and data are presented as the mean ± SD. For comparisons involving more than two groups, one-way analysis of variance (ANOVA) followed by an appropriate post hoc test was used. Comparisons between two groups (control and 10 μM EPA-treated groups) were analyzed using Student’s *t*-test. Oocyte and embryo developmental rates, including maturation rate, cleavage rate, and blastocyst rate, were analyzed using the chi-square test. Quantitative data obtained from qPCR and immunofluorescence analyses, including ROS levels, cortical granule distribution, mitochondrial distribution, and the proportion of apoptotic cells, were analyzed using Student’s *t*-test. All statistical analyses were performed using IBM SPSS Statistics 27 software (IBM Co., Armonk, NY, USA), and differences were considered statistically significant at *p* < 0.05.

## 3. Results

### 3.1. EPA at 10 μM Increased Porcine Oocyte Maturation and Parthenogenetic Embryonic Development

COCs tightly wrapped with more than three layers of cumulus cells were randomly divided into four groups corresponding to four concentrations of EPA (0, 1, 10 or 100 μM). After culturing for 42–44 h, oocyte maturation rates did not differ significantly between COCs treated with 1 μM and 100 μM EPA and the control group (*p* > 0.05) (Table 1). However, the oocyte maturation rate was significantly higher in the 10 μM EPA-treated group than in the control group (*p* < 0.01) (Table 1).

**Figure 1:**
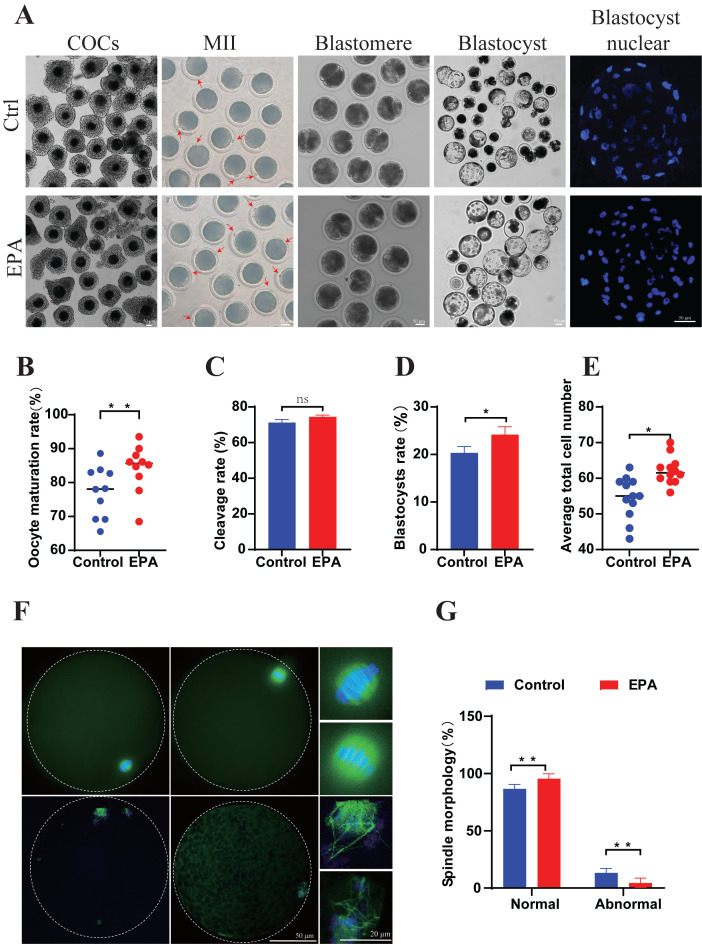
Treatment with 10 μM EPA improved porcine oocyte maturation and parthenogenetic embryonic development. (**A**) Images of COCs in vitro, MII oocytes (with arrows indicating the first polar bodies), two-cell blastomeres, blastocysts, and blastocyst nuclei derived from the control and 10 μM EPA-treated groups (scale bar = 50 μm). (**B**) Oocyte maturation rates in the control (*n* = 844) and 10 μM EPA-treated groups (*n* = 741) (N = 5). (**C**) Cleavage rates in the control (*n* = 257) and 10 μM EPA-treated groups (*n* = 246) (N = 10). (**D**) Blastocyst rates in the control (*n* = 257) and 10 μM EPA-treated groups (*n* = 246) (N = 5). (**E**) Average total cell number for blastocysts derived from the control (*n* = 50) and 10 μM EPA-treated groups (*n* = 50) (N = 12). (**F**) Spindle morphology of MII oocytes; normal and aberrant spindle morphologies are shown (scale bar = 50 μm), amplification area is shown in the box (scale bar = 20 μm). (**G**) Percentages of oocytes with normal and aberrant spindle morphologies derived from the control (*n* = 42) and 10 μM EPA-treated groups (*n* = 37) (N = 3). Means ± SD are shown. ** *p* < 0.01, * *p* < 0.05. N: replications, *n*: number of oocytes or embryos.

Based on the observed increase in oocyte maturation rates (*p* < 0.01), 10 μM EPA was utilized in subsequent analyses (Figure 1A,B). Parthenogenetic activation was subsequently conducted on control and 10 μM EPA-treated oocytes to investigate the effect of EPA treatment on embryonic development potential. The cleavage rate, blastocyst rate, and number of blastocyst cells were assessed (Figure 1A). The cleavage rate did not differ significantly (74.8 ± 1.72 vs. 71.2 ± 1.68, *p* > 0.05) (Figure 1C); however, the blastocyst rate (24.16 ± 1.21 vs. 20.3 ± 1.38, *p* < 0.05) (Figure 1D) and blastocyst cell count (62.17 ± 1.12 vs. 54.58 ± 1.70, *p* < 0.01) (Figure 1E) were significantly higher in the 10 μM EPA treatment group than in the control group. The results indicated that the in vitro nuclear maturation of porcine oocytes and subsequent embryonic development were significantly improved by 10 μM EPA treatment.

### 3.2. EPA at 10 μM Was Beneficial for the Cytoplasmic Maturation of Oocytes

**Figure 2:**
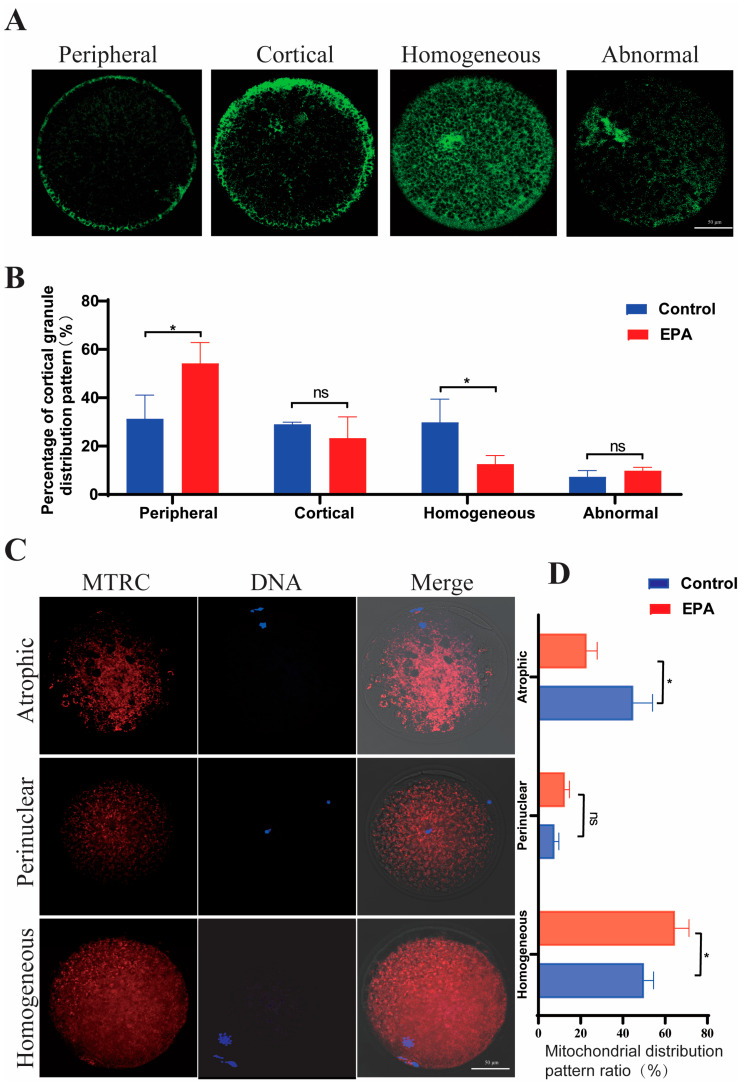
Treatment with 10 μM EPA improves porcine oocyte cytoplasmic maturation. (**A**) Distribution pattern of cortical granules in porcine MII oocytes, classified as peripheral, cortical, homogeneous, and abnormal (scale bar = 50 μm). (**B**) Percentages of peripheral, cortical, homogeneous, and abnormal distributions of cortical granules in the control (*n* = 44) and 10 μM EPA-treated groups (*n* = 35) (N = 3). (**C**) Distribution pattern of mitochondria in porcine MII oocytes, including homogeneous, perinuclear, and abnormal distribution patterns (scale bar = 50 μm). (**D**) Percentages of homogeneous, perinuclear, and atrophic mitochondria in the control (*n* = 39) and 10 μM EPA-treated oocytes (*n* = 38) (N = 3). Means ± SD are shown. * *p* < 0.05. N: replications, *n*: number of oocytes or embryos.

The effect of 10 μM EPA treatment on cytoplasmic maturation was evaluated. There was a reduction in the percentage of abnormal assembly from 13.35 ± 1.29% to 4.44 ± 1.40% (*p* < 0.01), indicating that 10 μM EPA improves spindle assembly significantly in porcine oocytes during in vitro maturation (Figure 1F,G). The distribution of cortical granules was classified as peripheral, cortical, homogeneous, and abnormal (Figure 2A). In comparison with the distribution in the control group, the administration of 10 μM EPA markedly increased the frequency of peripheral (32.19 ± 10.55% vs. 52.26 ± 11.98%, *p* < 0.01) and decreased the frequency of homogeneous (30.55 ± 8.63% vs. 16.58 ± 6.53%, *p* < 0.01), with no significant effect on the frequency of cortical granules (29.78 ± 11.12% vs. 11.48 ± 7.37%, *p* > 0.05) and abnormal distributions (7.48 ± 2.63% vs. 8.68 ± 1.19%, *p* > 0.05) (Figure 2B). Three mitochondrial distribution patterns were seen in MII oocytes: atrophic, perinuclear, and homogeneous (Figure 2C). The predominant pattern in MII oocytes was a homogeneous mitochondrial distribution, with a frequency of 64.6 ± 2.22% in the group treated with 10 μM EPA, significantly higher than that in the control group (50 ± 1.51%, *p* < 0.01) (Figure 2D). Moreover, in comparison with that in the control group, the frequency of an atrophic distribution was markedly lower in the 10 μM EPA group (22.86 ± 1.65% vs. 44.89 ± 3.04%, *p* < 0.01) (Figure 2D). These results indicate that the administration of 10 μM EPA can promote oocyte cytoplasmic maturation.

### 3.3. Treatment with 10 μM EPA Significantly Reduced Apoptosis in Cumulus Cells via RvE1

**Figure 3:**
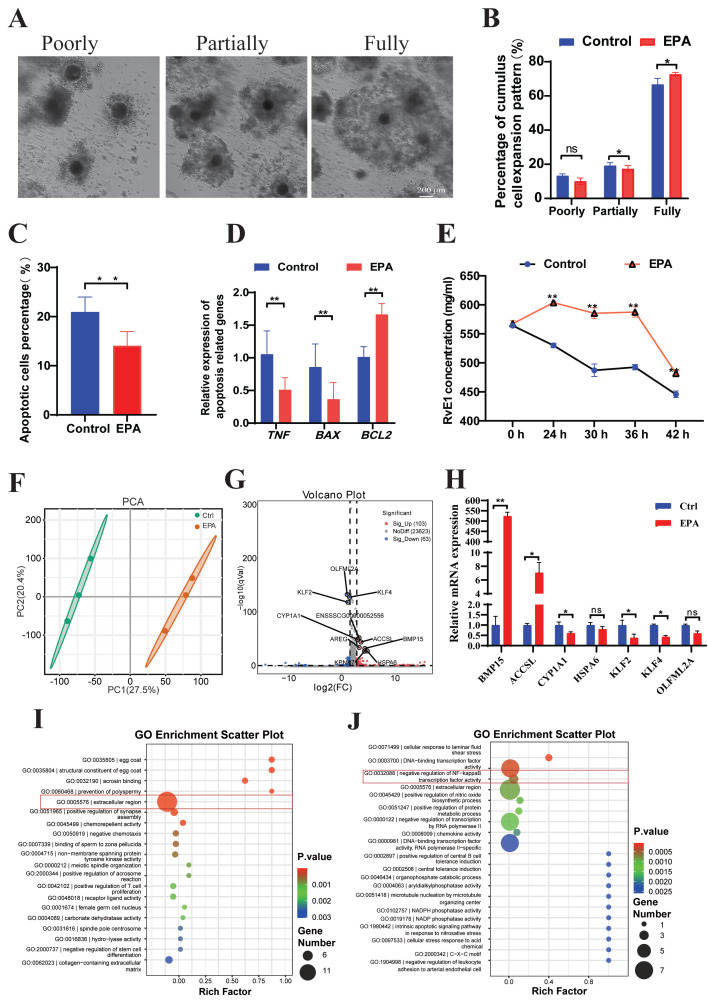
Treatment with 10 μM EPA significantly reduced cumulus cell apoptosis. (**A**) Morphology of COCs, classified as poor expansion, partial expansion, and full expansion (scale bar = 200 μm). (**B**) The percentages of poorly, partially, and fully expanded cumulus cells derived from the control (*n* = 193) and 10 μM EPA-treated groups (*n* = 189) (N = 3). (**C**) Percentages of apoptotic cumulus cells from the control and 10 μM EPA-treated groups (N = 3). (**D**) Relative expression of apoptosis-related genes, including TNF-α, BAX, and BCL2 in cumulus cells from the control and 10 μM EPA-treated groups. (**E**) Content of RvE1 in the culture media of the control and 10 μM EPA-treated groups at various time points, including 24, 30, 36 and 42 h (N = 3). (**F**) PCA plot of transcriptome data for the control and 10 μM EPA-treated cumulus cells. (**G**) Volcano plot of variance in gene expression based on fold changes and q-values, showing 103 significantly up-regulated genes and 63 significantly down-regulated genes in the EPA-treated group. (**H**) Relative expression levels of BMP15, ACCSL, CYP1A1, HSPA6, KLF2, KLF4, and OLFML2A. (**I**) GO enrichment analysis of up-regulated genes in 10 μM EPA-treated cumulus cells, colored by *p*-values. (**J**) GO enrichment analysis of down-regulated genes in 10 μM EPA-treated cumulus cells, colored by *p*-values. Means ± SD are shown. ** *p* < 0.01, * *p* < 0.05. N: replications, *n*: number of oocytes or embryos.

To determine the effect of 10 μM EPA on porcine oocytes, cumulus cell expansion was assessed after 42 h of culture (Figure 3A). The cumulus cell expansion was classified as poorly, partially and fully expanded. Treatment with 10 μM EPA increased the percentages of partially expanded COCs (19.92 ± 1.09% vs. 17.37 ± 0.63%, *p* < 0.05) and fully expanded COCs (66.7 ± 1.18% vs. 72.87 ± 0.33%, *p* < 0.05), with no significant effect on the percentage of poorly expanded COCs (13.38 ± 0.44% vs. 9.76 ± 0.62%, *p* > 0.05) (Figure 3B). The proportion of apoptosis in cumulus cells cultured for 42 h decreased significantly under 10 μM EPA treatment, as determined through flow cytometry (14.07 ± 2.89% vs. 20.93 ± 3.06%, *p* < 0.05) (Figure 3C). Additionally, real-time PCR results indicated that the relative expression levels of apoptosis-related genes (*TNF-α* and *BAX*) were dramatically reduced (*p* < 0.01), while the expression of the anti-apoptotic gene *BCL2* was markedly elevated (*p* < 0.01) in EPA-treated cumulus cells (Figure 3D). The levels of RvE1, the principal metabolite of EPA, were measured in the culture medium of COCs for both the control and EPA-treated groups at various time points. RvE1 levels at 24, 30, 36, and 42 h were all significantly higher than those in the control group (*p* < 0.01, Figure 3E).

Then, cumulus cells were collected for transcriptome sequencing to investigate the impact of 10 μM EPA treatment. Principal component analysis (PCA) revealed a clear separation between the two groups, indicating that EPA treatment induced a distinct global shift in the transcriptomic profile of oocytes compared with the control group (Figure 3F). The 10 μM EPA-treated group exhibited 103 significantly up-regulated genes and 63 significantly down-regulated genes (FC ≥ 1.5 or FC ≤ 0.67, *p* < 0.05) relative to levels in the control group (Figure 3G, Table S2). To confirm the validity of the transcriptome sequencing results, four up-regulated genes (*BMP15*, *ACCSL*, *CYP1A1,* and *HSPA6*) and three down-regulated genes (*KLF2*, *KLF4,* and *OLFML2A*) were evaluated using real-time PCR (Figure 3H). A GO enrichment analysis indicated that the up-regulated genes were enriched in terms pertaining to the extracellular region (Figure 3I, Table S3), while the down-regulated genes were enriched in categories pertaining to the negative regulation of NF-KappaB (Figure 3J, Table S3).

### 3.4. Treatment with 10 μM EPA Reduced Apoptosis in MII Oocytes

**Figure 4:**
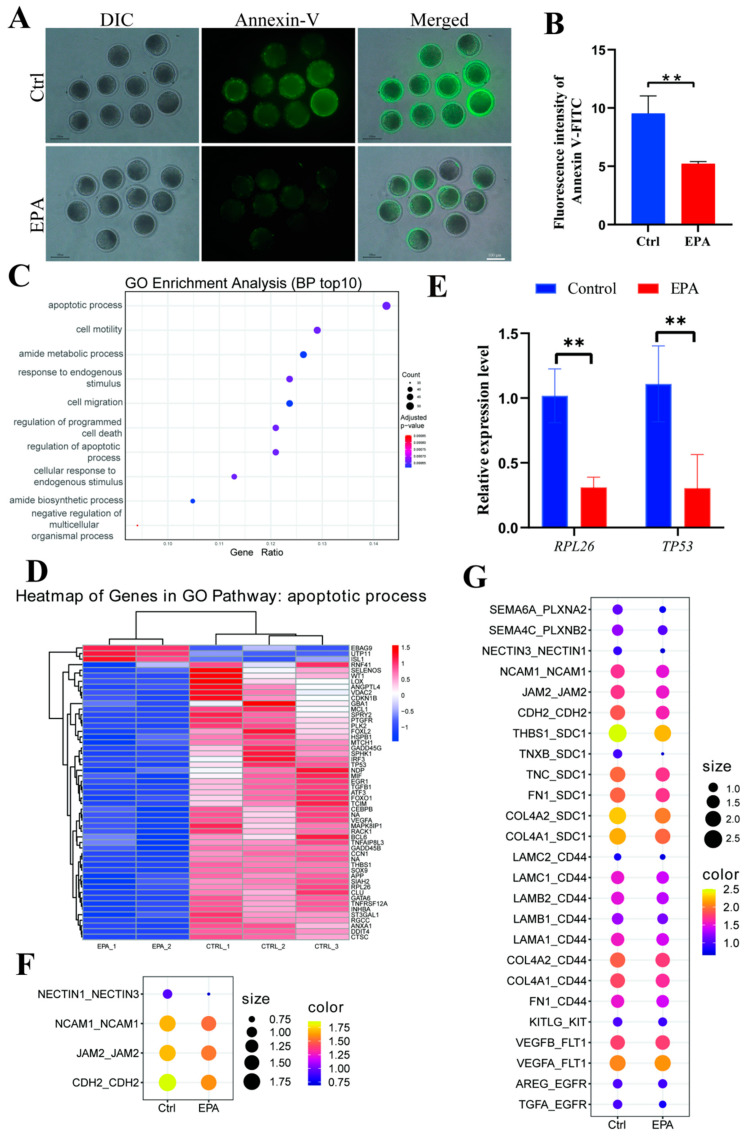
Treatment with 10 μM EPA reduced apoptosis in MII oocytes. (**A**) Early apoptosis in oocytes from the control and 10 μM EPA-treated groups, evaluated using Annexin V-FITC (scale bar = 100 μm). (**B**) Relative fluorescence intensity of Annexin V-FITC in MII oocytes derived from the control and 10 μM EPA-treated groups (N = 3). (**C**) GO enrichment analysis of significantly regulated genes in the 10 μM EPA-treated MII oocytes, where colors indicate *p*-values. (**D**) Heat map built using a panel of 50 down-regulated and 3 up-regulated genes in the 10 μM EPA-treated MII oocytes, enriched for GO terms related to the apoptotic process. (**E**) Relative expression levels of RPL26 and TP53. (**F**) Scatter plot showing ligand–receptor pair activity between cumulus cells and oocytes (receptor in oocytes and ligand in receptor). (**G**) Scatter plot showing ligand–receptor pair activity between cumulus cells and oocytes (receptor in cumulus and ligand in oocytes). Means ± SD are shown. ** *p* < 0.01. N: replications.

Early apoptosis in oocytes in the control and 10 μM EPA-treated groups was assessed using Annexin V-FITC (Figure 4A). The relative fluorescence intensity of Annexin V-FITC in MII oocytes from the 10 μM EPA-treated group was considerably lower than that of the control group (*p* < 0.05) (Figure 4B). To further understand the mechanism by which 10 μM EPA treatment influences the embryonic development of porcine oocytes, transcriptomes for 1500 oocytes in the control and EPA-treated groups were collected and sequenced. A PCA of transcriptome data revealed significant differences between the two groups (Figure S1), with 104 genes showing significant up-regulation and 768 genes showing significant down-regulation in the EPA-treated group (FC ≥ 1.5 or FC ≤ 0.67, *p* < 0.05) (Table S4). These differentially expressed genes were subjected to a GO functional enrichment analysis, revealing enrichment for terms related to the regulation of the apoptotic process (Figure 4C). 50 down-regulated and 3 up-regulated genes in the MII oocytes enriched for GO terms related to the apoptotic process (Figure 4D, Table S5). Real-time PCR results confirmed that the apoptosis-related genes *RPL26* and *TP53* were significantly down-regulated in the 10 μM EPA group (Figure 4E). Furthermore, in the groups treated with 10 μM EPA, we observed a significant decrease in ligand–receptor pair activity between cumulus cells and oocytes (Figure 4F,G).

### 3.5. Treatment with 10 μM EPA Reduced DNA Damage in MII Oocytes

**Figure 5:**
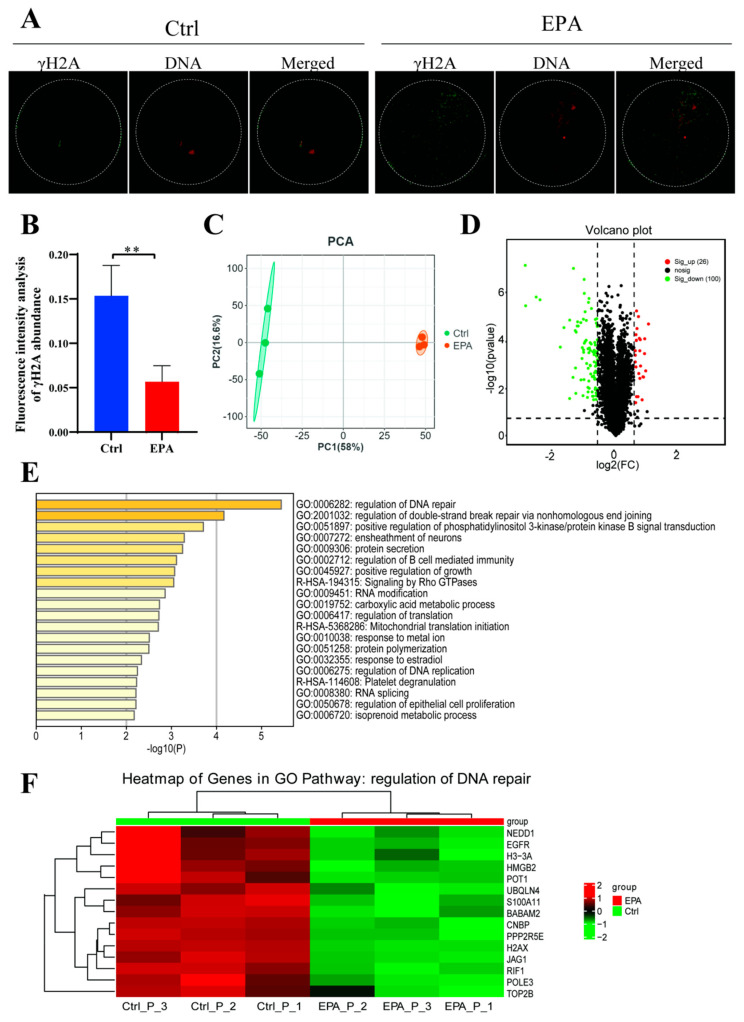
Treatment with 10 μM EPA reduced DNA damage in MII oocytes. (**A**) Images of DNA damage, evaluated through staining with the γ-H2AX antibody in control and 10 μM EPA-treated oocytes (scale bar = 10 μm). (**B**) γ-H2AX fluorescence intensity was evaluated in the control (*n* = 30) and 10 μM EPA-treated oocytes (*n* = 29) (N = 3). (**C**) PCA plot of proteome results for the control and 10 μM EPA-treated MII oocytes. (**D**) Volcano plot showing variance in proteins based on fold changes and q-values, with 26 significantly up-regulated proteins and 100 significantly down-regulated proteins in the EPA-treated group. (**E**) Pathway and process enrichment analysis of significantly downregulated proteins in the 10 μM EPA-treated MII oocytes, where colors represent *p*-values. (**F**) Heatmap built using a panel of 15 downregulated proteins in the 10 μM EPA-treated MII oocytes, annotated to GO terms related to the regulation of DNA repair. Means ± SD are shown. ** *p* < 0.01. N: replications, *n*: number of oocytes or embryos.

γ-H2AX staining was employed to quantify DNA damage in porcine MII oocytes (Figure 5A). The fluorescence intensity of γ-H2AX in control MII oocytes was substantially greater than that in MII oocytes treated with 10 μM EPA (*p* < 0.05, Figure 5B). For proteome sequencing, we collected 1500 oocytes from the control and 10 μM EPA treatment groups. Protein expression profiles for the two groups exhibited substantial differences, as indicated by a PCA (Figure 5C). In the 10 μM EPA-treated group, 26 proteins were significantly up-regulated and 100 proteins were significantly down-regulated (FC ≥ 1.5 or FC ≤ 0.67, *p* < 0.05) relative to levels in the control group (Figure 5D, Table S6). Differentially expressed proteins were evaluated through pathway and process enrichment analyses, revealing that the down-regulated proteins were involved in the control of DNA repair (Figure 5E). A heat map was constructed using a panel of 15 down-regulated genes in the 10 μM EPA-treated MII oocytes, annotated to GO terms related to the regulation of DNA repair (Figure 5F, Table S7).

### 3.6. Treatment with 10 μM EPA Reduced ROS Levels in MII Oocytes

**Figure 6:**
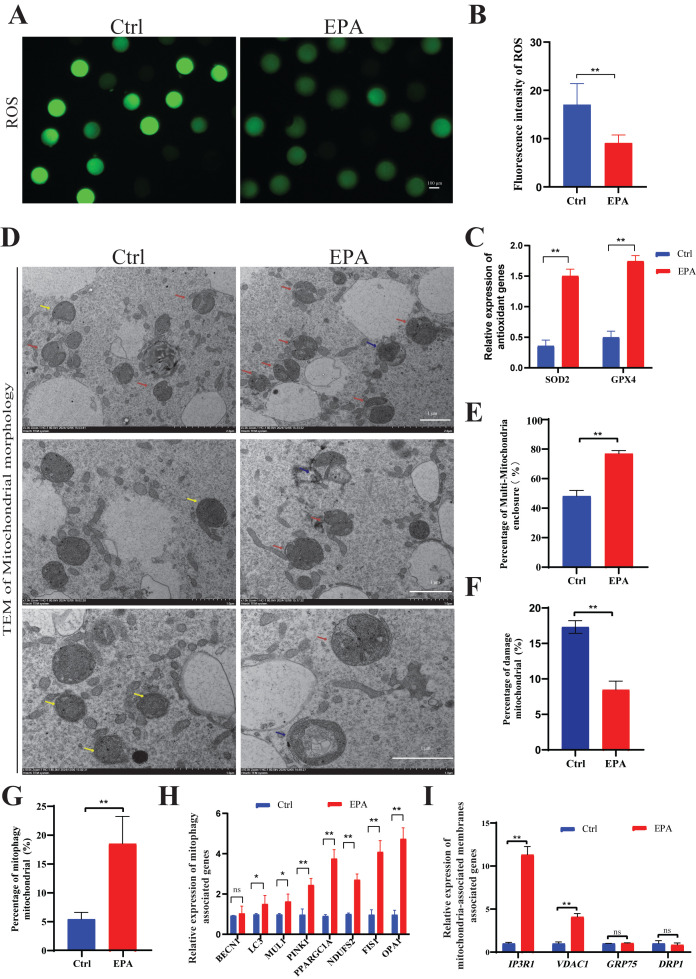
Treatment with 10 μM EPA reduced ROS levels in MII oocytes. (**A**) Reactive oxygen species levels in MII oocytes derived from the control and 10 μM EPA-treated group (scale bar = 100 μm). (**B**) Relative fluorescence intensity of reactive oxygen species in MII oocytes derived from the control and 10 μM EPA-treated groups (N = 3). (**C**) Relative expression levels of *SOD2* and *GPX4* in MII oocytes derived from the control and 10 μM EPA-treated groups (N = 3). (**D**) TEM images of mitochondrial morphology in the MII oocytes derived from the control and 10 μM EPA-treated group (red arrow, multiple intact mitochondria; blue arrow, mitophagy; yellow arrow, abnormal mitochondria) (scale bar = 1 μm). (**E**) Frequency of multi-mitochondria in MII oocytes derived from the control (*n* = 37) and 10 μM EPA-treated groups (*n* = 27) (N = 3). (**F**) Percentage of damage mitochondria in MII oocytes derived from the control (*n* = 37) and 10 μM EPA-treated groups (*n* = 27) (N = 5). (**G**) Percentage of mitophagy mitochondria in MII oocytes derived from the control (*n* = 37) and 10 μM EPA-treated groups (*n* = 27) (N = 3). (**H**) Relative expression levels of *PPARGC1A*, *NDUFS2*, *PINK1*, *LC3*, *FIS1*, *MUL1* and OPA1 in MII oocytes derived from the control and 10 μM EPA-treated groups (N = 3). (**I**) Relative expression levels of IP3R1, VDAC1, GRP75 and DRP1 in MII oocytes derived from the control and 10 μM EPA-treated groups (N = 3). Data are shown as means ± SD. * *p* < 0.05, ** *p* < 0.01. N: replications, *n*: number of oocytes or embryos.

We employed DCFH-DA staining to quantify intracytoplasmic ROS levels in porcine MII oocytes to evaluate the effect of 10 μM EPA treatment (Figure 6A). The control MII oocytes exhibited significantly higher ROS levels than those in the group administered 10 μM EPA (*p* < 0.01) (Figure 6B). To further explore the mechanism underlying this reduction in ROS levels, we examined the expression of key antioxidant genes. Notably, the expression levels of *SOD2* and *GPX4* were significantly upregulated in the 10 μM EPA-treated group (Figure 6C). Additionally, transmission electron microscopy revealed that 10 μM EPA treatment significantly increased the proportion of oocytes with multiple intact mitochondria (76.97 ± 4.37% vs. 48.24 ± 8.45%, *p* < 0.01) (Figure 6D,E) and reduced the incidence of necrotic mitochondria (8.48 ± 1.21% vs. 17.32 ± 0.90%, *p* < 0.01) compared to controls (Figure 6D,F). The frequency of mitophagic bodies was also elevated (*p* < 0.05), indicating enhanced mitochondrial turnover (Figure 6D,G). Mitochondrial physiology was modulated via upregulation of *PPARGC1A*, *NDUFS2*, *PINK1*, *LC3*, *FIS1*, *MUL1* and *OPA1*, indicating enhanced turnover, biogenesis, and energy metabolism with mitophagy (Figure 6H). Altered *IP3R1* and *VDAC1* expression and tighter endoplasmic reticulum (ER) contacts suggested strengthened Ca^2+^ signaling and metabolic coupling (Figure 6I).

## 4. Discussion

Previous studies have suggested that dietary supplementation with n-3 PUFAs is beneficial for female reproduction in various vertebrates [22]. In livestock, EPA-enriched diets or fish/algal oil supplementation during flushing, gestation, or lactation have been shown to improve follicular and uterine environments, reduce embryo loss, enhance offspring immunity and growth, and modify placental and milk fatty acid profiles [23,24,25,26]. Additionally, the concentration of EPA is markedly higher in larger follicles than in smaller follicles in humans [27], and EPA is also an integral component of mammalian oocytes [28]. Therefore, this study investigated the antioxidant regulatory effects of EPA during porcine oocyte in vitro maturation.

The EPA concentrations of 1, 10, and 100 µM were selected to span a low-to-high range commonly used in dose-dependent studies of n-3 PUFAs during oocyte in vitro maturation. Recent studies have demonstrated that DHA exerts beneficial effects within a restricted micromolar range by improving mitochondrial function, energy metabolism, and developmental competence, whereas higher concentrations are not considered functional doses [29,30]. Consistent with these reports, our initial screening indicated that 10 µM EPA enhanced nuclear maturation, while 100 µM showed no benefit and exhibited inhibitory trends (Table 1).

Accordingly, 10 µM EPA was selected as the working concentration based on its promotion of nuclear maturation, as indicated by the highest MII rate. At this concentration, EPA not only enhanced nuclear maturation but also improved mitochondrial function and redox homeostasis while suppressing apoptosis, indicating a coordinated improvement in overall oocyte quality rather than an isolated nuclear effect. Consistent with previous reports, these findings support the view that appropriate levels of n-3 PUFAs enhance oocyte developmental competence primarily through mitochondrial regulation and redox balance [30]. Building on this, our study demonstrated that the addition of 10 µM EPA to the in vitro maturation system improves porcine oocyte maturation and embryonic developmental ability significantly (Figure 1A–E). In this context, mitochondrial quality and redox homeostasis emerge as the central determinants of oocyte developmental competence, whereas other assessed parameters are best interpreted as downstream or supportive indicators of improved cellular homeostasis. Future studies will further refine EPA dosage using broader concentration ranges and additional functional endpoints.

The addition of 10 μM EPA significantly inhibits apoptosis in cumulus cells, possibly via the suppression of RvE1-mediated apoptotic signaling, consequently improving oocyte maturation efficiency. Cumulus cells contribute to both nuclear and cytoplasmic maturation through various mechanisms, including biochemical signaling, metabolic support, and structural interactions [31]. Our findings indicated that the 10 μM EPA-induced improvement in oocyte nuclear and cytoplasmic maturation may be attributed to reduced apoptosis and enhanced cumulus cell expansion. RvE1 in follicular fluid has been identified as a potential biomarker that enhances oocyte developmental competence by optimizing cumulus cell function [32]. Consistent with this, we observed that RvE1 levels in the COC culture medium were significantly elevated following EPA treatment at multiple time points, including 24, 30, 36, and 42 h (Figure 2F). Although the in vitro experiment revealed that RvE1 decreases the cumulus cell apoptotic rate and increases cell viability and proliferation, the underlying mechanism remains unclear [32]. RvE1 exerts anti-inflammatory, pro-resolving, and tissue-protective activities primarily through binding to its G protein-coupled receptor ChemR23 on various cell types [33]. Engagement of ChemR23 by RvE1 has been shown to attenuate TNF-α-mediated signaling cascades, thereby mitigating inflammatory responses in neurodegenerative diseases [34]. Our data showed that treatment with 10 μM EPA did not alter ChemR23 expression levels in cumulus cells (Figure S2). Instead, EPA supplementation reduced *TNF-α* expression in cumulus cells (Figure 3D,J), which may contribute to the observed inhibition of apoptosis, as cumulus cell-derived *TNF-α* has been reported to promote post-ovulatory oocyte aging in mice [35]. Together, these results indicate that EPA may improve cumulus cell viability by modulating the local inflammatory milieu. Importantly, improved cumulus cell function likely contributes to stabilization of the oocyte maturation microenvironment, which in turn enhances oocyte redox homeostasis and mitochondrial quality control. These coordinated improvements support proper spindle organization and cytoplasmic maturation, resulting in increased blastocyst formation and cell number.

In a healthy state, ROS and antioxidant levels remain balanced. Under a shift toward an overabundance of ROS, oxidative stress may lead to oocyte aging [36]. Excessive ROS accumulation is a major contributor to oxidative damage and apoptosis in oocytes, particularly during in vitro maturation [37]. Bearing unsaturated double bonds, EPA can be easily oxidized, thereby acting as an antioxidant compound that decreases the generation of ROS [38]. Consistent with this mechanism, the majority of DNA damage observed in oocytes has been attributed to apoptosis and ROS-induced oxidative stress [39]. In the present study, treatment with 10 μM EPA significantly reduced apoptosis (Figure 4A,B), DNA damage (Figure 5A,B), and ROS accumulation (Figure 6A,B) in oocytes, supporting its antioxidant role. Consistently, in MII oocytes, EPA not only decreased DNA damage and ROS accumulation but also upregulated the antioxidant enzymes SOD2 and GPX4, highlighting its role in enhancing the cellular redox defense system (Figure 6C). Collectively, these effects contribute to improved nuclear and cytoplasmic maturation, reduced apoptosis and inflammation, and optimized mitochondrial function, thereby enhancing overall oocyte quality.

In a healthy state, ROS and antioxidant levels remain balanced. Under a shift toward an overabundance of ROS, oxidative stress may lead to oocyte aging [36]. Excessive ROS accumulation is a major contributor to oxidative damage and apoptosis in oocytes, particularly during in vitro maturation [37]. Bearing unsaturated double bonds, EPA can be easily oxidized, thereby acting as an antioxidant compound that decreases the generation of ROS [38]. Consistent with this mechanism, the majority of DNA damage observed in oocytes has been attributed to apoptosis and ROS-induced oxidative stress [39]. In the present study, treatment with 10 μM EPA significantly reduced apoptosis (Figure 4A,B), DNA damage (Figure 5A,B), and ROS accumulation (Figure 6A,B) in oocytes, supporting its antioxidant role. Consistently, in MII oocytes, EPA not only decreased DNA damage and ROS accumulation but also upregulated the antioxidant enzymes SOD2 and GPX4, highlighting its role in enhancing the cellular redox defense system (Figure 6C). Collectively, these effects contribute to improved nuclear and cytoplasmic maturation, reduced apoptosis and inflammation, and optimized mitochondrial function, thereby enhancing overall oocyte quality.

Mitochondrial DNA is susceptible to ROS damage due to the absence of histone protection and an efficient DNA repair mechanism [40]. A previous study has reported that mitophagy is induced in porcine oocytes under toxin exposure [41]. Of note, the polyamine metabolite spermidine improves oocyte quality by enhancing mitophagy during female reproductive aging [42] and porcine oocyte in vitro maturation [43]. The fibrate drug bezafibrate protects against porcine post-ovulatory oocyte aging via antioxidant activity and mitochondrial protection [44]. Rapamycin, widely used as an inducer of autophagy, improves early embryonic developmental competence through protecting against apoptosis and controlling cellular quality mechanisms during in vitro embryo production [45,46]. Another study indicated that lack of NAMPT activity affects porcine oocyte maturation through its effects on mitochondrial function, spindle assembly, and lipid metabolism [47]. Consistent with previous results, high levels of mitochondrial damage were observed in porcine IVM oocytes, and enhanced mitophagy might explain the lower levels of mitochondrial damage under 10 μM EPA treatment (Figure 6D,G). Mitochondrial physiology was modulated via upregulation of *PPARGC1A*, *NDUFS2*, *PINK1*, *LC3*, *FIS1*, *MUL1*, and *OPA1*, indicating enhanced turnover, biogenesis, and energy metabolism with mitophagy (Figure 6H)**.** Additionally, in the context of oocyte maturation, the enclosure of multiple mitochondria by the ER is a specialized phenomenon linked to the unique energy, signaling, and structural requirements of early embryogenesis [48]. The high frequency of multiple mitochondria and high expression of genes linked to mitochondria-associated membranes (*IP3R1* and *VDAC1*) in MII oocytes may contribute to improvements in the embryonic development of oocytes treated with 10 μM EPA (Figure 6D,E,I). However, the mechanism by which EPA regulates mitochondrial morphology, ER–mitochondria interactions, and mitophagy in oocytes needs to be further investigated. The elevation of mitochondrial autophagy levels may be linked to the enhancement of embryonic developmental potential. These findings demonstrate the importance of EPA in governing mitochondrial morphology; however, further studies are needed to determine the exact regulatory mechanisms.

The present findings provide mechanistic insight into the role of EPA in oocyte maturation; however, their translational application in reproductive systems should be interpreted with appropriate context. The biological actions of EPA are sensitive to both dosage and exposure window and can vary with species, physiological stage, and background n-6:n-3 fatty acid balance, while excessive supplementation may perturb endocrine regulation and lipid homeostasis. Within this context, the EPA concentration employed for mechanistic analyses was selected based on its effect on MII maturation, and systematic dose–response studies incorporating multiple oocyte- and embryo-level endpoints will be required to delineate concentration-dependent effects and define appropriate application windows. Additionally, embryonic development was evaluated using a parthenogenetic activation model, which is suitable for assessing intrinsic oocyte developmental competence but does not encompass fertilization-dependent regulatory events. Moreover, blastocyst assessment relied primarily on developmental rate and total cell number, parameters that reflect overall developmental progression but may not capture more subtle qualitative attributes such as lineage specification or apoptotic status. Future studies will be required to validate these effects under fertilization-based embryo production systems and to further refine dose–response relationships using expanded embryo-quality endpoints.

## 5. Conclusions

Our findings demonstrate that EPA markedly enhances porcine oocyte maturation and embryonic developmental competence primarily by mitigating oxidative stress. EPA effectively reduces ROS accumulation, limits apoptosis, and preserves genomic integrity, while promoting mitochondrial quality control and improved organelle dynamics. These antioxidant-driven benefits highlight EPA as a valuable functional additive for optimizing in vitro oocyte culture systems. Beyond porcine reproduction, the robust cytoprotective effects of EPA suggest broader potential for improving assisted reproductive technologies in livestock production and possibly human fertility treatment, where safeguarding oocyte redox homeostasis is critical for reproductive success.

## Figures and Tables

**Table 1:** Effect of various concentrations of EPA on porcine oocyte in vitro maturation.

Group	No. of Replications	No. of COCs Cultured	No. of MII (% ± SD)
Control	7	657	536 (79.2 ± 2.84) ^c^
1 μM	7	606	482 (79.5 ± 2.94) ^c^
10 μM	7	598	524 (87.6 ± 1.19) ^a^
100 μM	7	592	470 (74.5 ± 2.97) ^c^

a, c Values with different superscript letters in the same column are significantly different (*p* < 0.01).

## Data Availability

The original contributions presented in this study are included in the article/Supplementary Information Files. Further inquiries can be directed to the corresponding authors.

## Supplementary Materials

The following supporting information can be downloaded at: https://www.mdpi.com/article/10.3390/antiox15010137/s1. Figure S1. PCA plot of transcriptome separating the control and 10 μM EPA-treated mature oocytes. Figure S2. Relative expression of ChmeR23 in the control and 10 μM EPA-treated cumulus cells. Table S1. Primers used for real-time PCR. Table S2. Differentially expressed genes in cumulus cells of the 10 UM EPA-treated group compared with the control group. Table S3. 3 down-regulated genes annotated with GO terms related to negative regulation of NF-kappaB transcription factor activity, and 13 up-regualted genes annotated with GO terms related to extracellular region. Table S4. Differentially expressed genes in MII oocytes of the 10 UM EPA-treated group compared with the control group. Table S5. Differentially expressed genes in MII oocytes (50 down-regulated and 3 up-regulated) related to the apoptotic process. Table S6. Differentially expressed proteins in the 10 μM EPA-treated MII oocytes compared with the control group. Table S7. 15 down-regulated genes annotated with GO terms related to the regulation of DNA repair.
