# Supplementary material for: Eicosapentaenoic Acid Improves Porcine Oocyte Cytoplasmic Maturation and Developmental Competence via Antioxidant and Mitochondrial Regulatory Mechanisms
Source: Antioxidants (Basel). 2026 Jan 21;15(1):137. doi: 10.3390/antiox15010137 (PMC12837691; doi:10.3390/antiox15010137)
Supplement: Supplementary file 1 [file antioxidants-15-00137-s001.zip › table S1.pdf]

Table S1 Primers used for real-time PCR

Gene ID	Symbol	Accession	Prime sequence
780421	CMKLR1	NM_001123100.1	Forward 5’-GCAGCTGGGACACATTTCCTG-3’<br>Reverse 5’-AAGCTCTCCAACGTGGGTCT-3’
403103	CYP1A1	NM_214412.1	Forward 5’-TATCCTCCGTTACCTGCCCA-3’<br>Reverse 5’-TGCGCCCCTTCTCAAAGATT-3’
100174959	KLF2	NM_001134351.2	Forward 5’-ACCGACGACGACCTCAACA-3’<br>Reverse 5’-CGTAGCAACTCCGACACCAA-3’
595111	KLF4	NM_001031782.2	Forward 5’-GGAGACGGAGGAGTTCAATGA-3’<br>Reverse 5’-GAGGAAGAGGATGAGGCTGAA-3’
448811	BMP15	NM_001005155.2	Forward 5’-GGACACTGCCTTCTTGTTACTC-3’<br>Reverse 5’-ATGCGATGCTGCCTGCTT-3’
100154062	OLFML2A	NM_001258360.2	Forward 5’-AGATGAACACGCTGGAAGAGA-3’<br>Reverse 5’-AGTGGTTCTCGTAGTGCTTCA-3’
396906	HSPA6	NM_001123127.1	Forward 5’-GCAGGCAGCAGTGTTGATG-3’<br>Reverse 5’-CCTCTGGATTAGCGTGGTCAT-3’
100521486	ACCSL	XM_021083162.1	Forward 5’-CTTCTCCATCGCCACTTCC-3’<br>Reverse 5’-GCCTCCTTGTCCAATTCCTCT-3’
397454	IP3R1	XM_021069280.1	Forward 5’-CAGATGGTGGACAACTCAGGC-3’<br>Reverse 5’- GGCTTTGGAACTCGTGGCAGA-3’

397010	VDAC1	XM_005652948.3	Forward 5’-AGAAGATGGCTGTGCCTCCT-3’<br>Reverse 5’- CACTTTGGTGGTCTCCGTGT-3’
100521183	GRP75	XM_005661695.3	Forward 5’-AAGTAGGGCAGGGGAAGCTA-3’<br>Reverse 5’- AGGCCACAGGGTAGCTAGAA-3’
100624497	DRP1	XM_021092060.1	Forward 5’-TGCCTCAGATCGTAGTGGTG-3’<br>Reverse 5’- GCTTCAACTCCATTTTCTTCTCC-3’
100514904	MUL1	XM_003356176.5	Forward 5’-TTCCACCCCTCCATCCAGTCC-3’<br>Reverse 5’-CCCCCACCTTCAGCATCTCCT-3’
397086	TNF-α	NM_214022.1	Forward 5’-CAACGGCGTGAAGCTGAAAGA-3’<br>Reverse 5’-CTGATGGTGTGAGTGAGGAAA-3’
396633	BAX	XM_003127290.5	Forward 5’-CCCGAGAAGTCTTTTTCCGAG-3’<br>Reverse 5’-GAAGTCCAGCGTCCAGCCCAT-3’
100049703	BCL2	XM_021099593.1	Forward 5’-TCGGTGGGGTCATGTGTGTGG-3’<br>Reverse 5’-GCAGGTGCCGGTTCAGGTACT-3’
100627662	RPL26	NM_001243458.1	Forward 5’-CAATCCTTTTGTGACCTCCGA-3’<br>Reverse 5’-CAGTTGTGCCATTAGCCTTCT-3’
397276	TP53	NM_213824.3	Forward 5’-TTTCACCCTCCAGATCCGTG-3’<br>Reverse 5’-TTCAGCTCCAAGGCGTCATT-3’
733576	BECN1	XM_013980932.2	Forward 5’-AGGAGCTGCCGTTGTACTGT-3’<br>Reverse 5’-CACTGCCTCCTGTGTCTTCA-3’

100322893	LC3	NM_001190290.1	Forward 5’-GCCTCTCAGGAGACTTTCGG-3’<br>Reverse 5’-GAGCTCCGTTTTTCTGCGTG-3’
100515613	PINK1	XM_021095478.1	Forward 5’-GGGCTCTCCAACCCCTTTTAT-3’<br>Reverse 5’-GCCTCTTGCTGGCATCTCGCT-3’
396823	GAPDH	NM_001206359.1	Forward 5’-GCCATCACCATCTTCCAGG-3’<br>Reverse 5’-CACGCCCATCACAAACAT-3’
