# Supplementary material for: Eicosapentaenoic Acid Improves Porcine Oocyte Cytoplasmic Maturation and Developmental Competence via Antioxidant and Mitochondrial Regulatory Mechanisms
Source: Antioxidants (Basel). 2026 Jan 21;15(1):137. doi: 10.3390/antiox15010137 (PMC12837691; doi:10.3390/antiox15010137)
Supplement: Supplementary file 1 [file antioxidants-15-00137-s001.zip › Table S3.pdf]

Table S3 3 down-regulated genes annotated with GO terms related to negative regulation of NF-kappaB transcription factor activity, and 13 up-regualted genes annotated with GO terms related to extracellular region.

Term	Description	LogP	Log(q-value)	Symbols
GO:0032088	negative regulation of NF-kappaB transcription factor activity	-3.26228	-1.4285	KLF4, FOXJ1, DDIT3
GO:0005576	extracellular region	-4.12762	-1.93062	ZP2, ZP3, ZP4, CFAP45, LAMA4, GHSR, OOSP2, BMP15, TMPRSS11F, OOSP3, NRG1, CXCL14, MSMB
