# Supplementary material for: Eicosapentaenoic Acid Improves Porcine Oocyte Cytoplasmic Maturation and Developmental Competence via Antioxidant and Mitochondrial Regulatory Mechanisms
Source: Antioxidants (Basel). 2026 Jan 21;15(1):137. doi: 10.3390/antiox15010137 (PMC12837691; doi:10.3390/antiox15010137)
Supplement: Supplementary file 1 [file antioxidants-15-00137-s001.zip › Table S7 .pdf]

Table S7 15 down-regulated genes annotated with GO terms related to the regulation of DNA repair.

Term	Description	LogP	Log(q-value)	Symbols
GO:0006282	regulation of DNA repair	-5.434754647	-1.091	EGFR, H2AX, TOP2B, BABAM2, POT1, RIF1, UBQLN4, S100A11, POLE3, HMGB2, CNBP, H3-3A, PPP2R5E, NEDD1, JAG1
