# Supplementary figures and images for: Eicosapentaenoic Acid Improves Porcine Oocyte Cytoplasmic Maturation and Developmental Competence via Antioxidant and Mitochondrial Regulatory Mechanisms
Source: Antioxidants (Basel). 2026 Jan 21;15(1):137. doi: 10.3390/antiox15010137 (PMC12837691; doi:10.3390/antiox15010137)

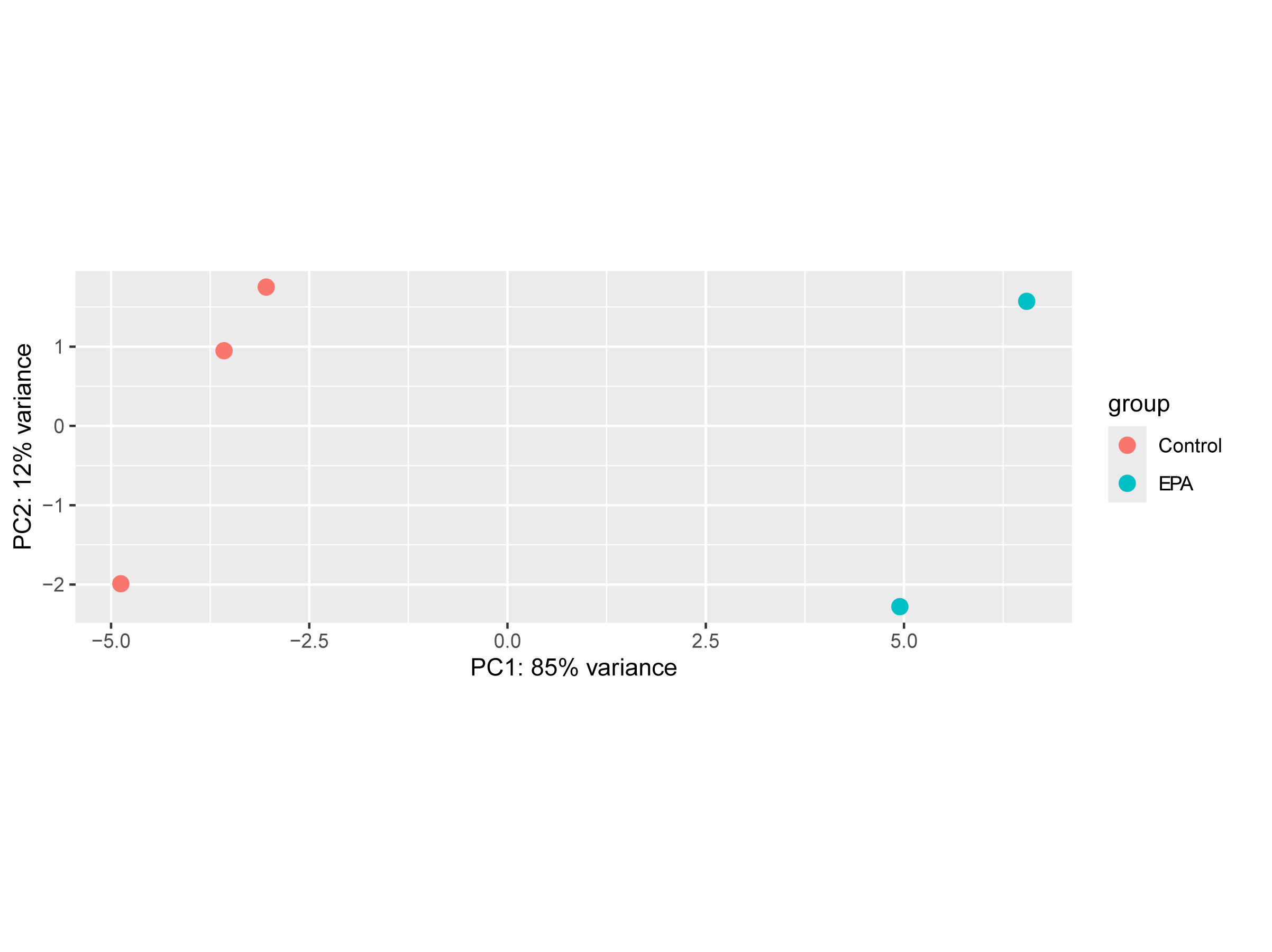

Supplement: Supplementary file 1 [file antioxidants-15-00137-s001.zip › Figure S1.tif]

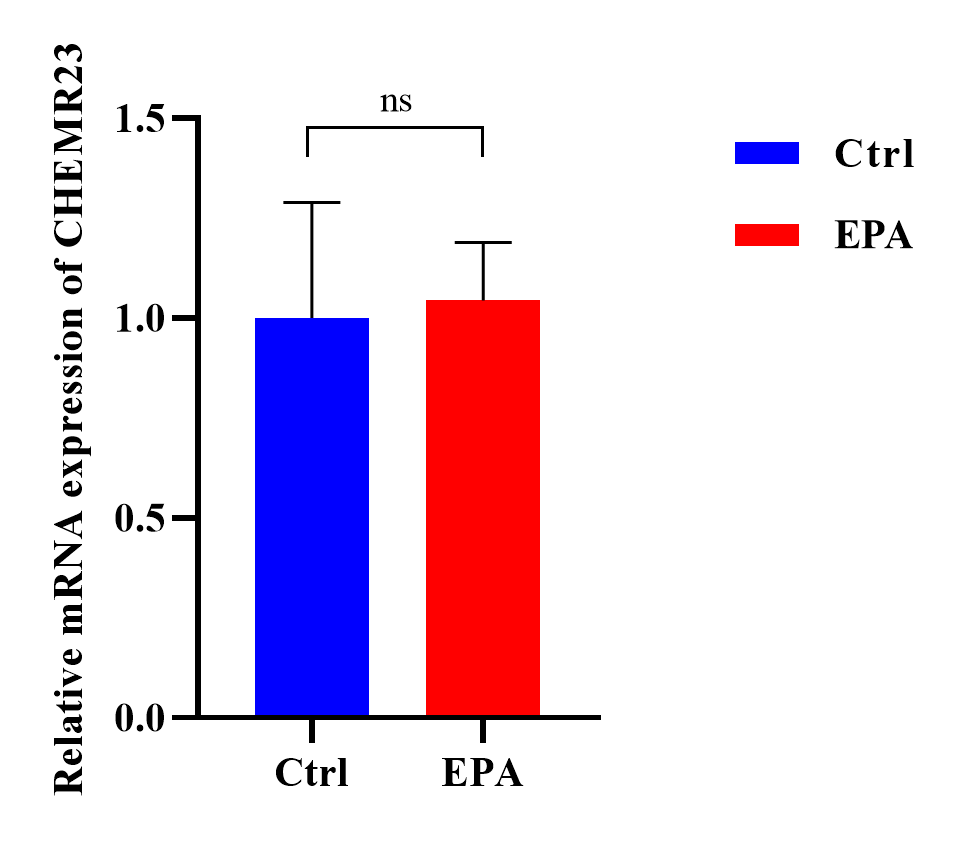

Supplement: Supplementary file 1 [file antioxidants-15-00137-s001.zip › Figure S2.tif]
